# Evolutionary tradeoffs, Pareto optimality and the morphology of ammonite shells

**DOI:** 10.1186/s12918-015-0149-z

**Published:** 2015-03-07

**Authors:** Avichai Tendler, Avraham Mayo, Uri Alon

**Affiliations:** Department of Molecular cell biology, Weizmann Institute of Science, Rehovot, 76100 Israel

**Keywords:** Multi-objective optimality, Repeated evolution, Pareto front, Diversity, Performance, Goal

## Abstract

**Background:**

Organisms that need to perform multiple tasks face a fundamental tradeoff: no design can be optimal at all tasks at once. Recent theory based on Pareto optimality showed that such tradeoffs lead to a highly defined range of phenotypes, which lie in low-dimensional polyhedra in the space of traits. The vertices of these polyhedra are called archetypes- the phenotypes that are optimal at a single task. To rigorously test this theory requires measurements of thousands of species over hundreds of millions of years of evolution. Ammonoid fossil shells provide an excellent model system for this purpose. Ammonoids have a well-defined geometry that can be parameterized using three dimensionless features of their logarithmic-spiral-shaped shells. Their evolutionary history includes repeated mass extinctions.

**Results:**

We find that ammonoids fill out a pyramid in morphospace, suggesting five specific tasks - one for each vertex of the pyramid. After mass extinctions, surviving species evolve to refill essentially the same pyramid, suggesting that the tasks are unchanging. We infer putative tasks for each archetype, related to economy of shell material, rapid shell growth, hydrodynamics and compactness.

**Conclusions:**

These results support Pareto optimality theory as an approach to study evolutionary tradeoffs, and demonstrate how this approach can be used to infer the putative tasks that may shape the natural selection of phenotypes.

**Electronic supplementary material:**

The online version of this article (doi:10.1186/s12918-015-0149-z) contains supplementary material, which is available to authorized users.

## Background

**Figure 1:**
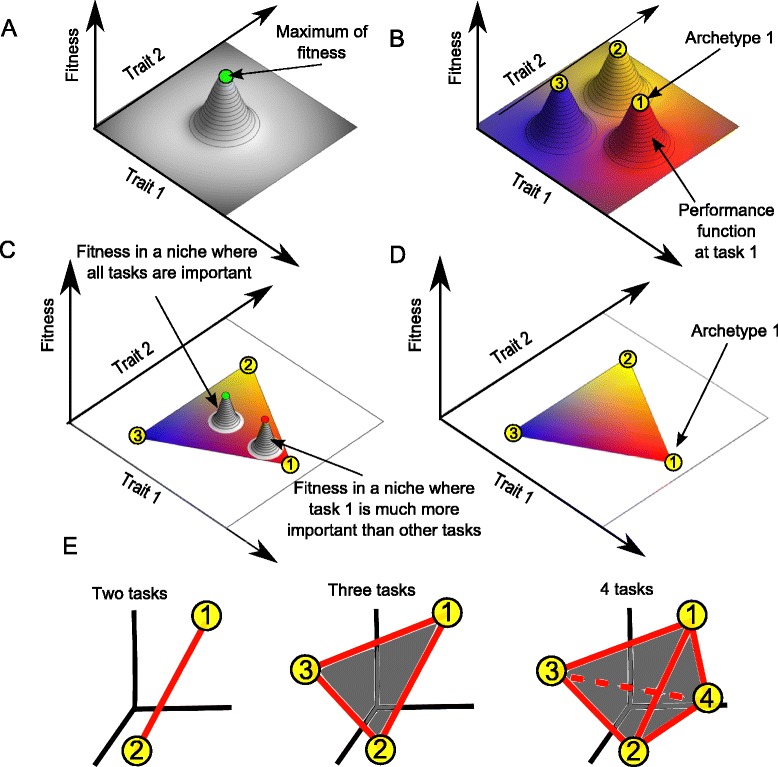
**An overview of Pareto theory for evolutionary tradeoffs. (A)** The classical viewpoint of a fitness landscape: phenotypes are arranged along the slopes near the peak of a fitness hill maximum. **(B)** In contrast, the Pareto viewpoint suggests a tradeoff between tasks. For each task there is a performance function, which is maximal at a point known as the archetype for that task. The fitness function in each niche is a combination of the different performance functions (in general, fitness is an increasing function of performances, possibly a nonlinear function). **(C)** Optimality in a niche in which task 1 is most important, is achieved near archetype 1 (red maximum). Optimality in a niche in which all tasks are equally important, is achieved close to the middle of the Pareto front (green maximum). **(D)** The entire Pareto front- the set of maxima of all possible fitness functions that combine these performances- is contained within the convex hull of the archetypes. **(E)** Different numbers of tasks give various polygons or polyhedra, generally known as polytopes. Two tasks lead to a suite of variation along a line segment. Three tasks lead to a suite of variation on the triangle whose vertices are the three archetypes. Four archetypes form a tetrahedron. This is true while there are enough traits measured: in lower dimensional trait spaces one finds projections of these polytopes.

Organisms that need to perform multiple tasks face a fundamental tradeoff: no phenotype can be optimal at all tasks [1-8]. This tradeoff situation is reminiscent of tradeoffs in economics and engineering. These fields analyze tradeoffs using Pareto optimality theory [9-13]. Pareto optimality was recently used in biology to study tradeoffs in evolution [2,5-8,14]. In contrast to the classic fitness-landscape approaches in which organisms maximize a single fitness function [15], the Pareto approach deals with several performance functions, one for each task, that all contribute to fitness (Figure 1A-B).

Pareto theory makes strong predictions on the range of phenotypes that evolve in such a multiple-objective situation: the evolved phenotypes lie in a restricted part of trait-space, called the Pareto front. The Pareto front is defined as phenotypes that are the best possible compromises between the tasks; phenotypes on the Pareto front can’t be improved at all tasks at once. Any improvement in one task comes at the expense of other tasks.

Shoval et al. [14] calculated the shape of the Pareto front in trait space under a set of general assumptions. Evolved phenotypes were predicted to lie in a polygon or polyhedron in trait space, whose vertices are extreme morphologies, called archetypes, which are each optimal at one of the tasks (Figure 1B-D). Thus, two tasks lead to phenotypes on a line that connects the two archetypes, three tasks to a triangle, four tasks to a tetrahedron and so on (Figure 1E). These polyhedra can have slightly curved edges in some situations [16]. One does not need to know the tasks in advance: tasks can be inferred from the data, by considering the organisms closest to each archetype. This theory can be rejected in principle by datasets which lie in a cloud without sharp vertices, and hence do not fall into well-defined polygons.

The Shoval et al. theory has been applied so far to datasets from animal morphology [14,17], bacterial gene expression [14,18], cancer [19], biological circuits [20] and animal behavior [21]. In all of these cases, multi-dimensional trait data was found to be well described by low-dimensional polygons or polyhedra (lines, triangles, tetrahedrons). Tasks were inferred based on the properties of the organisms (or data-points) closest to the vertices. An algorithm for detecting polyhedra in biological data and inferring tasks was recently presented [19].

However, some of the fundamental predictions of the theory have not been tested yet. The theory predicts that as long as the tasks stay more-or-less constant, for example dictated by biomechanical constraints, the vertices of the polygon also do not change. Moreover, the polygons in the theory are not necessarily due to phylogenetic history, but rather to convergent evolution to Pareto-optimal solutions. Thus, for example, after a mass extinction which removes most of the species from a class [22,23], survivor species are predicted to evolve to re-fill the same polygon as their ancestors [22,24].

To test these predictions requires a class of organisms evolving over geological timescales, with mass extinctions, and whose geometry is well-defined and can be linked to function. An excellent model system for this purpose is ammonoid fossil shells.

**Figure 2:**
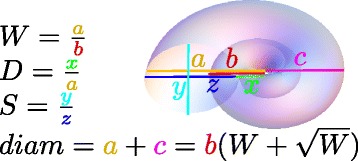
**Raup morphospace coordinates.** Ammonoid shell morphology can be described by three dimensionless geometrical parameters: W, the whorl expansion rate, is defined by a/b in the figure. D, the internal to external shell ratio, is x/a. S, the opening shape parameter, is y/z. The shell diameter can also be related to the parameters in this figure as shown.

Ammonoids were a successful and diverse group of species, which lived in the seas from 400 to 65 million years ago (mya). Ammonoid shells can be described by a morphospace defined by three geometrical parameters, defined in the pioneering work of David Raup (Figure 2) [15,25,26]. In this morphospace, the outer shell is a logarithmic spiral, whose radius grows with each whorl by a factor W, the whorl expansion rate. There is a constant ratio between the inner and outer shell radii, denoted D. Finally, the shell cross section can range from circular to elliptical, as described by S, the third parameter. Raup’s W-D-S parameterization can be robustly measured from fossils [26] although the coiling axis changes throughout ontogeny and thus, the coiling axis is sometimes difficult to exactly locate in actual specimens [27,28]. It has been the setting for extensive research on ammonoid morphology and evolution [22,29-32], as well as the morphology of other shelled organisms [33,34].

**Figure 3:**
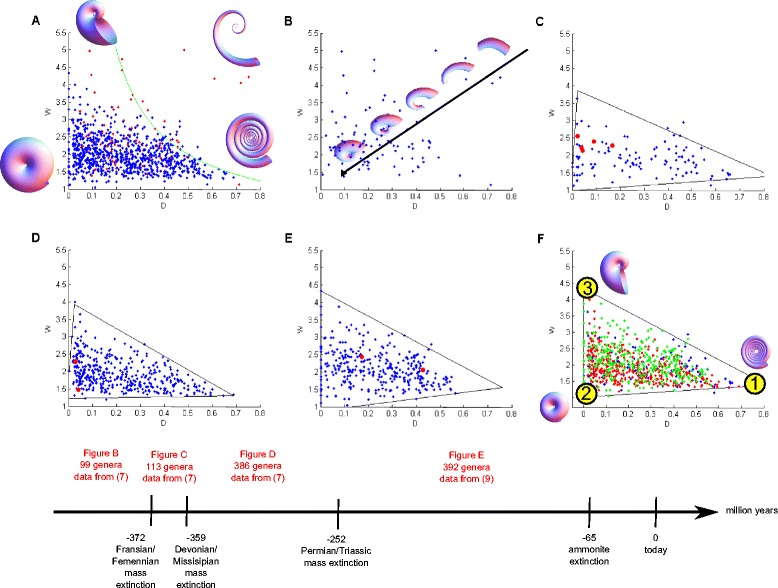
**Ammonoids repeatedly filled the same triangle in D-W plane after mass extinctions. (A)** All of the ammonoid data used in the present study. Red points are genera before the FF (first) mass extinction, genera after FF are denoted by blue points. The green curve is W = 1/D. **(B)** Ammonoids before the FF extinction, together with a schematic arrow for the direction of evolution from ancestral taxa. **(C)** Genera between FF and DM mass extinctions fill out a triangle (obtained by applying the SISAL algorithm [41] on the dataset), surviving genera from the FF mass extinction are denoted by red bold points. **(D)** Ammonoids between DM and PT mass extinctions fill a triangle, surviving genera from the DM mass extinction are denoted by red bold points. **(E)** Ammonoids after the PT mass extinction fill a triangle, surviving genera from the PT mass extinction are denoted by red bold points. **(F)** Ammonoids from different periods, together, genera between FF and DM are denoted blue, DM to PT in red and post PT in green. The shell morphologies of the three archetypes at the vertices of the triangle are shown.

Plotting each genus of ammonoids as a point in this morphospace, ignoring coiling axis changes, Raup discovered that most of the theoretical morphospace is empty: many possible shell forms are not found. The existing forms lie in a roughly triangular region in the W-D plane (Figure 3A). One reason for this distribution is geometric constraints. Researchers have suggested that ammonoids tend to lie to the left of the hyperbola W = 1/D [15,26], because beyond this curve shells are gyroconic (shells with non-overlapping whorls) (Figure 3A upper right corner). Such gyroconic shells are mechanically weaker and less hydrodynamically favorable [35,36]. It is noteworthy, however, that shells to the right of the curve do exist in nature, for example in the Bactritida or Orthocerida lineages, which are probably ancestral to the ammonoids (Figure 3B, top right) [37-40], as well as in heteromorph ammonoids that occasionally occur in the Mesozoic and more commonly in the Cretaceous. Thus the W = 1/D curve is unlikely to be an absolute geometric constraint (for more evidence, see Additional file 1).

Studies in recent years have considered a larger dataset of ammonoids than Raup [22,29,30]. Work, Saunders and Nikolaeva [22] show that after each mass extinction, ammonoid genera refill the same roughly triangular morphospace [24]. The repeated convergence to the same suite of variation raises the question of the relation between ammonoid morphology and function. Most studies hypothesize a fitness function, which has an optimum in the middle of the triangular region [15,35,36] (Figure 1A). The fitness function is often taken to be dominated by hydrodynamic drag; this assumption is compelling since the contours of hydrodynamic efficiency, experimentally measured by Chamberlain [35], show peaks at positions close to the most densely occupied regions of morphospace [15]. The ammonoid genera are assumed to also occupy the slopes that descend from the fitness peaks, until bounded by the geometric constrains [15].

Interestingly, Raup did not espouse the idea of a single task (such as hydrodynamic efficiency) dominating fitness, but rather noted that multiple tasks might be at play [26]. In every niche, different tasks become important, leading to niche-dependent fitness functions with different maxima (Figure 1B-D). The idea of multiple tasks was elegantly employed by Westermann [42], who described ammonoid morphospace by mapping it to a triangle. At the vertices are three ‘end member’ morphologies which correspond to three lifestyles. Each morphology is mapped to a point in the triangle, which is interpreted as portraying the relative distance from the end members and hence the relative weights of the three lifestyles. The Westermann morphospace was useful in comparing different datasets and in interpreting ammonoid lifestyles [43,44]. The main drawback of the Westermann morphospace is that, because it involves nonlinear dimensionality reduction, different morphologies can be mapped to the same point, and in some cases slight differences in shape can lead to large differences in the Westermann projection. Thus, it is of interest to seek a relation between shape and tasks without such drawbacks.

**Figure 4:**
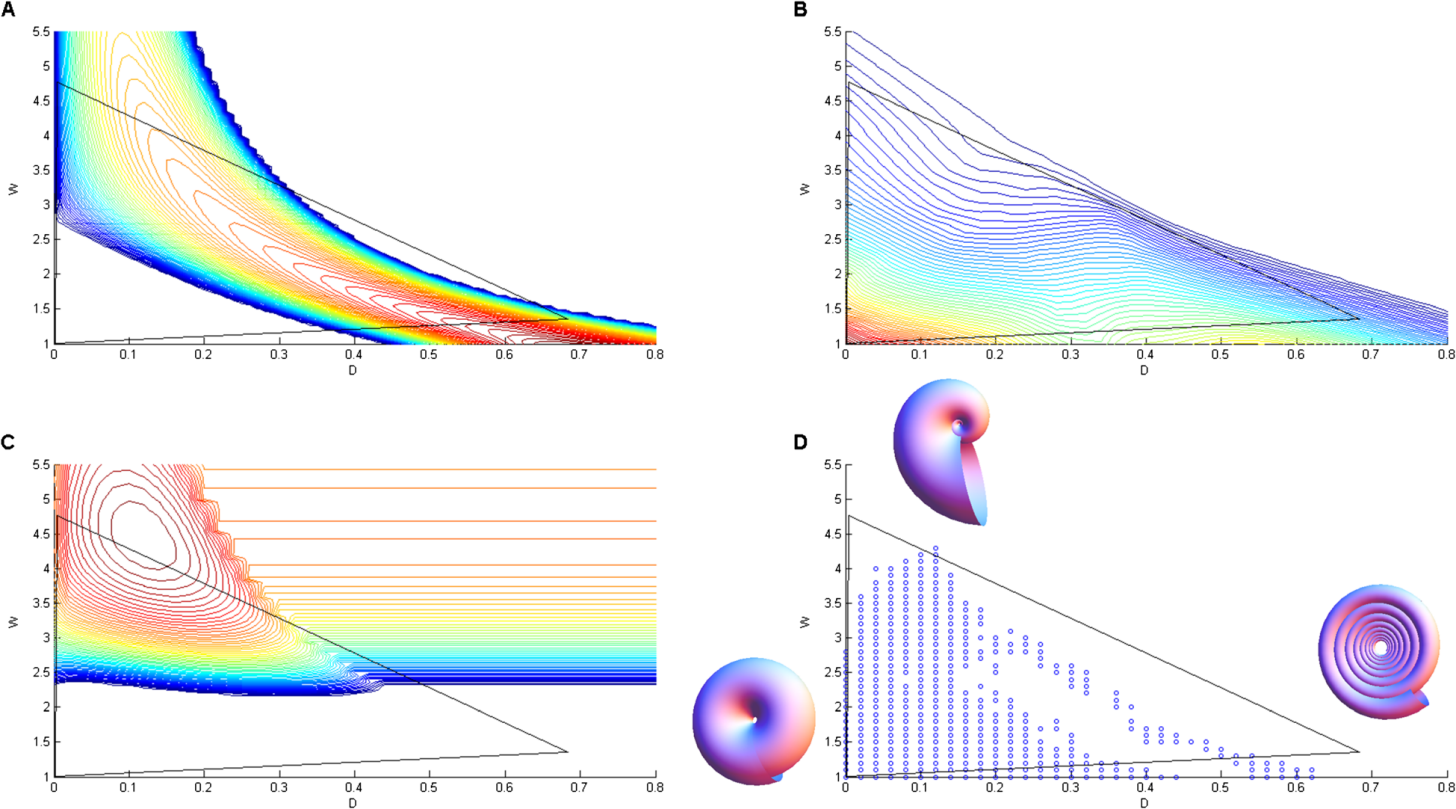
**The performance contours of the three putative tasks for ammonoid shells. (A)** Contours for shell economy, defined as the ratio of internal volume to shell volume, with red denoting high values, and blue low values. For gyroconic shells (non-overlapping whorls), this performance function becomes constant, and equal to the lowest contour shown (deep blue). The triangle encapsulating the entire ammonoid dataset is shown in black. **(B)** Contours for the drag coefficient measured by Chamberlain [36], red lines denote lower drag or better hydrodynamics. **(C)** Contours for the growth function defined in the main text, red lines denote quicker growth. **(D)** The contours of the three tasks give rise to a suite of variation denoted by blue points.

To address this, we explore evolutionary tradeoffs between tasks in the framework of Pareto optimality theory, to quantitatively explain the suite of variation in direct morphospace (without dimensionality reduction), and to infer the putative tasks at play. We find that ammonoid morphology in the W-D-S morphospace falls within a square pyramid, suggesting five tasks. The triangular region observed by Raup is the projection of this pyramid on the W-D plane, and the Westermann morphospace is a dimensionality reduction of the three-dimensional pyramid to a two-dimensional triangle. We propose putative tasks whose performance contours jointly lead to the observed suite of variations, including hydrodynamic efficiency, shell economy, compactness and rapid shell growth. The position of each species in this pyramid, namely its distance from each vertex, indicates the relative importance of each task in the niche in which that species evolved. After the FF and DM mass extinctions (Fransian/Femennian and Devonian/Missisipian 372 and 359 mya), surviving ammonoids refill essentially the same pyramid. After the PT extinction (Permian/Triassic 252 mya), part of the pyramid is refilled. These findings lend support to the Pareto theory of evolutionary tradeoffs in the context of evolution on geological timescales.

## Results

### Ammonoid distributions in the W-D plane converge to a similar triangle after major extinctions

We begin by considering ammonoid morphology in the W-D plane, and later consider the three dimensional W-D-S space (Figure 3). We combine the data of Saunders, Work and Nikolaeva [22] for Paleozoic ammonoids (598 genera, before the PT mass extinction- for extinction timeline see Figure 3 lower panel), with the data of McGowan [29] for Mesozoic ammonoids (392 genera, after PT). The data is classified into three sets between mass extinctions: from FF to DM (113 genera, Figure 3C), from DM to PT (386 genera, Figure 3D), and after PT (392 genera, Figure 3E).

We tested whether the ammonoid distribution in each set falls in a triangle more closely than randomized data, based on the statistical test of [14]. We use an archetype analysis algorithm (SISAL) [45] to find triangles, which enclose as much of the data as possible. We find that a triangle describes each dataset much better than randomized datasets in which the W and D coordinates are randomly permuted (see Methods). Randomized data rarely fill out a triangle as well as the real data (*p* = 0.02 for FF-DM data and *p* = 0.01 for the DM-PT and post PT sets).

We next tested how similar the triangles are for the three datasets. We computed the ratio between the intersection area of the triangles to the union area as a measure for triangle similarity. The three triangles show large ratios of intersection to union area (0.84, 0.74 and 0.71 for the (FF-DM, DM-PT), (FF-DM, post PT) and (DM-PT, post PT) pairs respectively, *p* <10^-4^ compared to randomly generated triangles, see Methods), indicating that the triangles are very similar.

We conclude that after each extinction, ammonoids re-populate essentially the same triangular region. The vertices of the triangle describing the joint dataset of ammonoids after FF (Figure 3F) are

1 $$ \left({D}_1^{*},{W}_1^{*}\right)=\left(0.69,1.35\right)\sim \left(0.7,1.35\right) $$

2 $$ \left({D}_2^{*},{W}_2^{*}\right)=\left(0.003,1.04\right)\sim \left(0,1\right) $$

3 $$ \left({D}_3^{*},{W}_3^{*}\right)=\left(0.004,4.59\right)\sim \left(0,4.6\right) $$

We next ask which tasks might relate to each of the vertices.

### Economy of shell material may determine the first archetype

Raup [26] suggested that a possible need of the ammonoids is to maximize their internal volume relative to shell volume. This is important if shell production is costly, and also in terms of buoyancy considerations. Ammonoids are thought to achieve neutral buoyancy by balancing shell weight with buoyancy from their air-filled chambers; high internal volume relative to shell material extends the range over which neutral buoyancy can be reached [46,47].

To calculate shell material relative to internal volume at each point in morphospace, we follow Raup and assume that shell thickness is a fixed fraction of radius, namely thickness/radius = 0.077, as measured by [47]. Interestingly, this ratio is close to the optimal ratio of thickness/radius =0.07 from calculations of mechanical strength in tube-like bones [4]. We improve slightly on Chamberlin and Raup’s original calculation [48] by numerically evaluating the necessary integrals rather than using the analytical approximations of [49] (see Methods), yielding corrections of about 10%.

The maximum of internal volume relative to shell thickness occurs at (*D*_1_, *W*_1_) = (0.67, 1). This point is close to archetype one $\left({D}_1^{*},{W}_1^{*}\right)=\left(0.7,1.35\right).$ The calculated contours of internal volume relative to shell thickness-namely the performance contours of the task of economy- have a curving ridge that points towards the third archetype (Figure 4A). Performance drops sharply on either side of this ridge.

**Figure 5:**
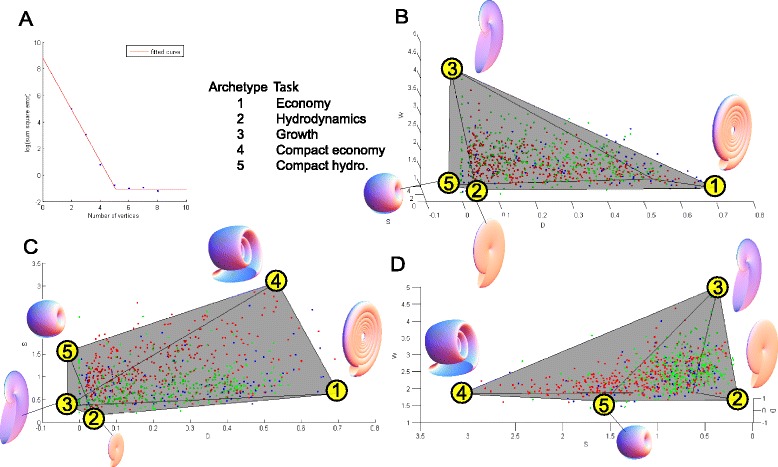
**The three dimensional Pareto front of the ammonoid dataset. (A)** The RMS error for PCHA optimal polygons and polyhedra is computed for different numbers of possible vertices: line, triangle, tetrahedron, 5-vertex polyhedron, etc. Error decreases with increasing the number of archetypes up to 5. Increasing the number beyond 5 doesn't improve the fit by much (for more evidence for the pyramidal shape of the data, see Additional file 1). **(B-D)** The best fit 5-archetype polygon resembles a square pyramid. Blue points denote FF to DM ammonoids, red are DM to PT and green are post PT ammonoids. Archetypes are numbered, their morphology is shown, and the suggested tasks are listed in panel A.

### The second archetype may optimize hydrodynamics

We conjecture that the second archetype maximizes the hydrodynamic efficiency of the ammonoids. Low drag is important for ammonoids in order to swim rapidly. Hydrodynamic efficiency is measured by the drag coefficient, which is a dimensionless number specific to each geometrical shape.

The drag coefficient is proportional to the force which should be applied in order to keep an object of a given surface area moving at a given velocity in water. Drag coefficients were measured by Chamberlain [36] using plexiglass models of shells [50].

The contours of hydrodynamic efficiency are shown in Figure 4B. Drag monotonically increases with D and W, hence we can conclude that the ammonoid morphology with minimal drag has the lowest possible values of D and W, namely (*D*_2_, *W*_2_) = (0, 1) This is close to the second vertex of the triangle, archetype two at $\left({D}_2^{*},{W}_2^{*}\right)=\left(0.003,1.04\right)$.

### The third archetype may optimize rapid shell growth

The remaining vertex of the triangle, archetype 3, has a large value of W. Thus the shell radius at this vertex increases rapidly with each revolution of the spiral (evolute morphology). There are different possible tasks that might relate to large W, including rapid growth, shell-orientation and swimming capabilities. In Westermann morphospace, large W compared to D and S is interpreted as nektonic (actively swimming). Here, we wish to demonstrate an essential approach, and thus focus on one of these potential tasks: rapid growth, and leave other possibilities to future study.

The fossil dataset we use does not contain information on growth. However, if we assume that the ability to generate shell material (hence to grow) is proportional to body mass (see [51] Chapter 16, but also [52,53]) we can predict the growth, or at least a function proportional to growth, using only the dimensionless parameters we have. An evolute shell allows volume to grow rapidly with each whorl. Rapid growth may be important because predation tends to decrease with organism size. This would select for increased W. However, the whorl expansion rate W cannot grow without bound in order to avoid cyrtoconic shells- the shell must close over itself at least once to provide space for the ammonoid body (with possible exceptions such as heteromorphs which go beyond the present discussion). A coiled shell is also important in order to benefit from increased shell thickness, because until the ammonoid is closed, the thinner shell is exposed to the outside. The small value of D is also reasonable for such a function, because when W is large, a small D is a must in order to benefit from the advantages of W < 1/D (see Additional file 1 for a more detailed explanation).

A similar function was suggested in snails where shell growth rate was found to be larger in snails in the presence of predators [54].

To be concrete, we consider a putative performance function that penalizes the ammonoid for the smallness of its diameter, namely ${P}_3={\displaystyle \underset{0}{\overset{\infty }{\int }}\frac{1}{diam(t)}dt}$ (see Methods). Contours of this performance function are shown in Figure 4C. The function peaks at (*D*_3_,*W*_3_) = (0.12,4.44) close to the third archetype $\left({D}_3^{*},{W}_3^{*}\right)=\left(0,4.6\right).$ At this archetype, ammonoids reach large diameters most rapidly.

One may ask if the advantage of growth comes from the increased diameter which might make the ammonoid too large for specific predators, or from the increased shell thickness which make it stronger. It is difficult to distinguish between this two conjectures since from [47] we know that this quantities are proportional to one another. It is likely that both diameter and shell thickness contribute to fitness.

The three putative performance functions, shell economy, hydrodynamic efficiency, and shell growth together give rise to a triangular shaped Pareto front. The Pareto front boundaries are given by the points of tangency of the contours of the different performance functions. Figure 4D shows the computed Pareto front, which resembles a slightly curved triangle, and is similar to the observed suite of variation.

### Ammonoid data is enclosed by a pyramid in W-D-S morphospace

Up to now, we considered ammonoid morphology projected on the W-D plane. We now turn to the analysis of the data in the three-dimensional morphospace, given by W,D and S—whorl expansion, radii ratio and the shape of the shell opening. Low values of the parameter S correspond to oblate elliptical openings, giving rise to compressed shells (Figure 5B, front). An S value of 1 corresponds to a circular shell opening; high values corresponding to depressed shell morphologies (Figure 5B, rear).

**Figure 6:**
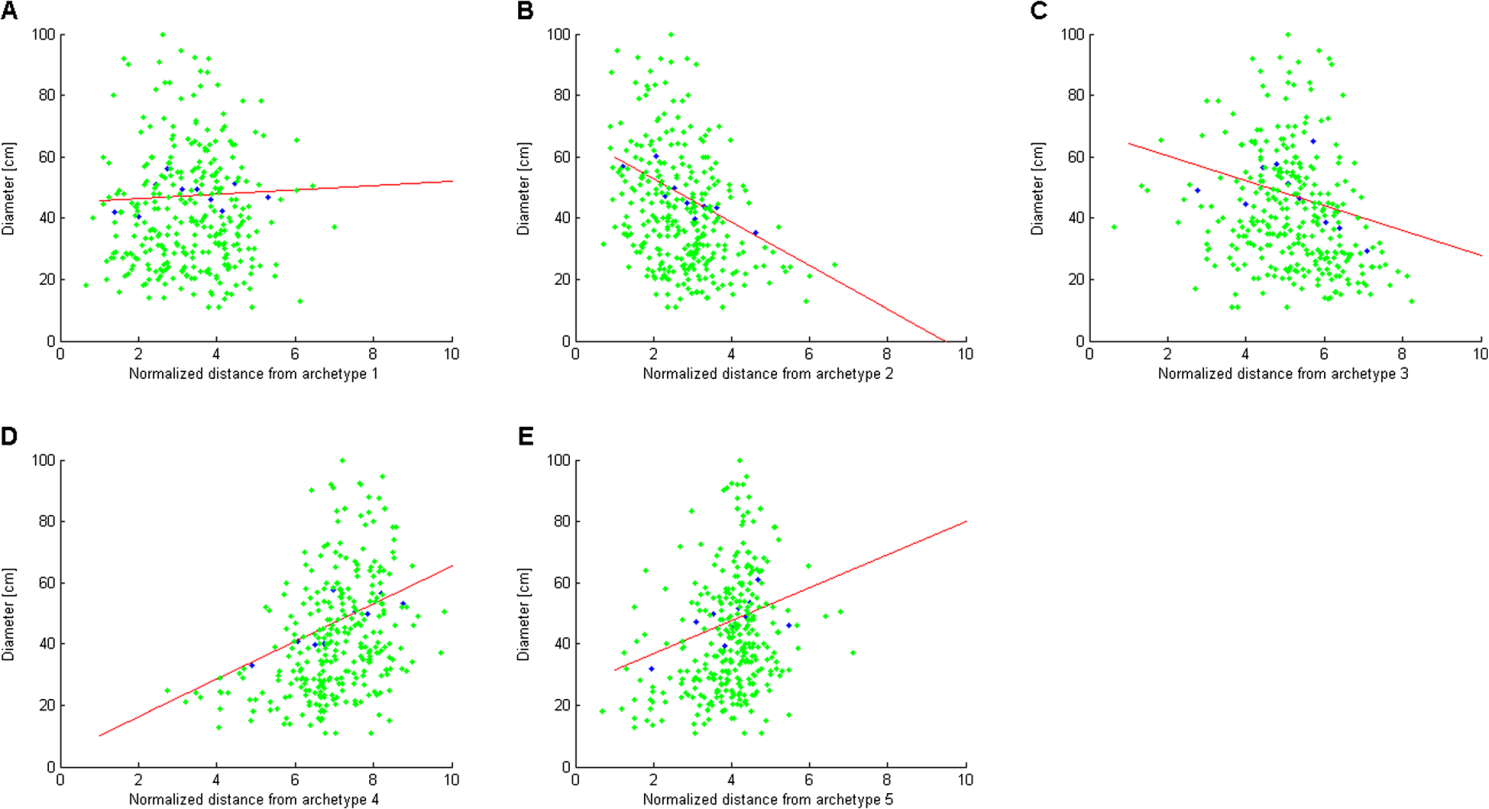
**Size is enriched at some of the archetypes.** Ammonoid shell diameter as a function of distance from each archetype shows that small diameters are prevalent near archetypes 4 and 5. Data includes diameter for 392 genera (green points) [29], divided into 10 bins with equal number of genera according to the distance from each archetype. Average diameter for each bin is plotted (blue points). For convenience, a fit of the averages to a line is shown. **(A)** No diameter enrichment near archetype 1 (p = 0.29). **(B)** Positive diameter enrichment near archetype 2 (*p* <10^-4^) **(C)** Positive diameter enrichment near archetype 3 (p = 0.0007). **(D)** Negative diameter enrichment near archetype 4 (*p* <10^-4^) **(E)** Negative diameter enrichment near archetype 5 (p = 0.0002).

We attempted to enclose the 3D dataset by polygons with 2 to 8 vertices. We evaluated the extent to which each polygon explains the data, by calculating the RMS distance of points outside the polyhedron. We find that beyond 5 vertices, the RMS error does not decrease significantly (Figure 5A): Shapes with 6 or more vertices do not improve the closeness of fit appreciably. Hence a 5-vertex polygon is a parsimonious description of the data (Figure 5B-D). This 5-vertex shape has four vertices that lie approximately on a plane. We thus consider this shape as a pyramid. A square pyramid encloses the data better than randomly permuted dataset with *p* <10^-4^ (see Methods).

The five vertices of the pyramid suggest five archetypes, whose coordinates are given in Table 1. The square base of the pyramid has two vertices at low S (vertices 1 and 2), and two others, which match them for W and D values, but have higher S values (vertices 4 and 5, respectively). The apex of the pyramid has a thin opening with S = 0.3.

**Table 1:** **Summary of suggested ammonoid archetypes**

**Suggested task**	**W**	**S**	**D**	**Archetype number**
Economy of shell material	1.3	0.7	0.65	1
Hydrodynamic drag	1.55	0.2	0.04	2
Shell growth	4.6	0.3	0	3
Compactness + economy	1.6	3.2	0.5	4
Compactness + hydro.	1.07	1.8	0.01	5

Coordinates of the archetypes found by the archetype analysis algorithms with 5-archetypes, along with their putative tasks.

Projecting the pyramid on the W-D plane, we find that the apex of the pyramid matches the ‘growth’ archetype described above; the ‘economy’ and ‘hydrodynamic’ archetypes each corresponds to the projection of two 3D archetypes: the economy archetype corresponds to archetypes 1 and 4, and the hydrodynamic archetype to archetypes 2 and 5 (Figure 5).

### Economy, hydrodynamic and growth performance functions are maximized near three of the pyramid vertices

We repeated the calculation of economy performance (ratio of internal volume to shell thickness) in three dimensions. The 2D contours shown previously (Figure 4A) were evaluated at S = 1. By varying S, we find that the maximal economy is found at (*D*_1_, *S*_1_, *W*_1_) = (0.67, 1.01, 1). This is reasonably close to vertex 1 of the observed pyramid $\left({D}_1^{*},{S}_1^{*},{W}_1^{*}\right)=\left(0.65,0.69,1.35\right).$The internal volume to shell volume ratio in this vertex is 96% of the optimum value. For comparison, this ratio drops to nearly zero near vertices 2 and 5 of the pyramid.

The hydrodynamic efficiency measured in [36] includes data at values of S other than S = 1. This indicates that optimal hydrodynamic efficiency is at low S values, i.e. *S*→0. The resulting optimum is thus close to vertex 2 of the pyramid, which is $\left({D}_2^{*},{S}_2^{*},{W}_2^{*}\right)=\left(0.03,0.19,1.55\right):$ note the low values of D,S and W.

Archetype 3 has an S value close to 0.3. The dependence of the growth performance function on S comes only implicitly through the volume-to-surface ratio. It is unclear from the present simplified model for the growth performance function why 0.3 (and not 1) is selected as the optimal S value for archetype 3. This S value might be due, for example, to diminishing returns of shell production per body mass. In other words, the assumption that shell material production is constantly proportional to body mass might be imprecise. If shell production grows slower than linearly with body mass (as supported by [52,53]), this will favor smaller-volume ammonoids with smaller value of S that will increase diameter faster.

### The last two pyramid archetypes may be related to size

Two pyramid vertices remain to be explained, vertices 4 and 5. These vertices have large values of S, and correspond to depressed shells (Figure 5B-D). We find that these shapes have the smallest ratio of surface area to volume (as detailed in Additional file 1). They are therefore the most globular in the suite of variation, in the sense that their height is most similar to their width and depth.

One feature of globular ammonoids is small size for a given internal volume, because spherical shapes have the minimal diameter of all shapes with the same volume. Up to now, we did not consider the absolute size of the ammonoids, only on dimensionless shape traits W, D and S. To address this, we correlated data by McGowan [29] on ammonoid size (diameter) with distance from the five vertices of the pyramid. We find an enrichment of small ammonoids most strongly near archetypes 4 and 5: the genera nearest to these vertices have the smallest diameters (Figure 6). Archetypes 2 and 3 are enriched with large ammonoids and archetype 1 has weak enrichment since its S value (which is related to globularity, Additional file 1) is relatively larger than archetypes 2 and 3 (Table 1). Archetypes 4 and 5 may thus correspond to economy and hydrodynamic tasks respectively, combined with a need for smallness. This relation between diameter and globularity is in line also with [55], which used a different dataset.

**Figure 7:**
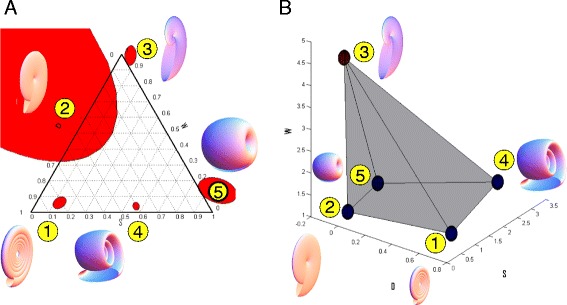
**Archetypes in trait space and in Westermann space. (A)** Ammonoids in Westermann space. The red ellipses are the pyramid archetypes projected on the Westermann space, with 5% error in the archetype positions. Three of the pyramid archetypes lies near the vertices of the triangle, another archetype is near the edge because both D and S are dominant. The region near archetype 2 is severely warped (large red ellipse) because D,S and W are all relatively small. (note that archetypes 1,3 and 4 are inside the corresponding ellipses) **(B)** The pyramid in the W-D-S trait space, the ellipsoids are 5% errorbars around each vertex. These small ellipsoids translates to the red ellipses of subfigure A when switching to Westermann morphospace.

We further compared the way ammonoids from different periods fill out the pyramid. The main difference between periods is between Paleozoic and Mesozoic genera. Mesozoic ammonoids tend to have lower S values than Paleozoic ones, as found by McGowan [29]. In the pyramid, they are more densely arrayed near the face defined by vertices 1, 2 and 3, and away from 5 and especially from 4. This may be interpreted in the present framework as a shift in the niches occupied by later ammonoids, in which tasks corresponding to archetypes 4 and 5 contribute less to fitness than they did in the Paleozoic niches.

Finally, we mapped the five archetypes of the pyramid to the Westremann morphospace. We find that that three archetypes, 1, 3 and 5, map near the three vertices of the Westermann triangle (serpenticone, oxycone and sphericone, respectively). The two other vertices of the pyramid map closer to the edges of the triangle. Some of the archetypes map slightly outside of the triangle since they are exptrapolated points which lie outside of the ammonite dataset. We also asked about the sensitivity of this transformation, by testing a small region around each archetype (a sphere of radius 5% of the total variation in each coordinate). We find that one of the archetypes, archetype 2, lies in a region of morphospace which is severely warped by the Westermann transformation, and maps to a wide region in the triangle. The other archetypes are less sensitive and map to relatively small regions of the triangle (Figure 7).

## Discussion

This study explored how tradeoffs between multiple tasks may have contributed to the evolution of ammonoid shell morphology. Ammonoid shell data on 990 genera were studied in Raup’s three parameter morphospace. The data is well described by a square pyramid. This finding is interpreted in light of Pareto theory on tradeoffs between tasks. The five vertices of the pyramid may be interpreted as archetype morphologies optimal for a single task, and morphologies in the middle of the pyramid are generalists which compromise between the tasks.

We propose candidate tasks for the archetypes. Hydrodynamic efficiency is a good candidate for one of these tasks, and is maximized near vertex 1 of the pyramid (low W,D and S). Other putative tasks can be inferred from the position of the other vertices of the pyramidal shell distribution. We propose that economy of shell material (perhaps related to buoyancy) is a second task, quantitated by the ratio of internal volume and total shell material. The maximum of this function matches one of the vertices of the pyramid. A third task may be rapid growth. A performance function relating to rapid growth of ammonoid diameter is maximal near the apex of the pyramid, at shells with high W. Two other tasks may relate to small spherical-like shells combined with low drag and high economy.

It is interesting to relate this study with previous work by Westermann and Ritterbush based on the idea that ammonoids face tradeoffs between different tasks, which determine their morphologies [42-44]. Westermann proposed a morphospace which, instead of working in D-W-S space, works in a 2-dimensional projection which consists of ratios of related measurements. Westermann morphospace has many advantages. As a method to reduce 3-dimensional data into 2-dimensional one, it helps visualize data in order to achieve better understanding of the geometry. It is also useful in understanding the different niches that ammonoids occupy and infer the various tasks they face [43,44].

Westermann's 2-dimensional representation also has drawbacks. As a dimensionality reduction method, it loses information about the data. Ammonoid shells with very different geometries can be mapped to the same point in Westermann morphospace. Moreover, because the Westemann map is nonlinear, there are regions in morphospace that map to the triangle with relatively large errors. For example, a small region around the point of minimal values of D, S and W (which is close to the pyramid archetype 2, which we relate to low drag) can mapped to the entire Westermann triangle (linked to what seen in Figure 7) depending on slight variation in the values.

The present approach does not show these drawbacks because it works directly in W-D-S morphospace. It thus distinguishes between morphologies which are mapped to the same Westermann point. The square pyramid identified here suggests two new tasks (or end members) in addition to Westermanns three. These are archetypes 2 and 4 which correspond to hydrodynamic drag and compact shell economy. We also propose a different interpretation of the other three tasks. For example the Westermann oxycone endmember, which is linked to nektonic lifestyle, corresponds to our archetype 3 (which relates to rapid growth). Furthermore, the same oxyconic endmember relates to certain morphologies near our archetype 2 (which relates to low drag, similar to the task suggested for this endmember in Westermann morphospace).

The present study also bears on the question of geometric constraints in evolution [15]. The W = 1/D line in ammonoids is thought to be an outstanding example of a geometric constraint [15], because of the disadvantages of the open shell morphology beyond this curve. This assumption is challenged by the existence of organisms with W > 1/D, including lineages ancestral to ammonoids as well as several heteromorphs [56]. The present approach can make the concept of geometric constraint more precise by relating it to biological tasks. We consider the performance functions of tasks, some of which indeed show a decline beyond the W = 1/D line. In particular, economy and hydrodynamics contours both begin to sharply decline when W > 1/D (Figure 4). This provides a more principled explanation, replacing strict geometric constraint with the more subtle dependence of specific performance functions on geometry. Other taxa may perform a different set of tasks, including a task with an archetype in the ‘forbidden for ammonoid’ region, W > 1/D. Such tasks might explain the morphology of the taxa which show gyroconic shells. An alternative view is that some characters states do not require a functional explanation, but rather were neutral enough for a clade to succeed for some time.

This study adds to previous studies that used the Pareto approach to analyze other biological systems [14]. These systems showed lines, triangles or tetrahedra in morphospace. Ammonoids are the first system in which a pyramidal Pareto front is observed. For this purpose, we find that the archetype analysis algorithm PCHA [45] is an efficient way to detect high order polyhedra in data [19].

The present approach can be readily extended to other shelled organisms such as gastropods and bivalves. One application of the present approach is a quantitative inference of which task is important for fitness in the particular niche of each genus. The closer the shell morphology is to a given vertex of the pyramid, the more important the corresponding task. Since ammonoid shells are carried by currents and found in rocks far from the habitat of the living organism, it is challenging to connect morphology with behavior. The present approach can offer quantitative inference about the relative contribution of tasks to fitness, to provide insight into the ecological niche of these extinct organisms. More generally, this study supports basic predictions of the Pareto theory for evolutionary tradeoffs [14], which we hope will be useful also for other biological contexts.

## Conclusions

This study supports fundamental predictions of the Pareto theory of tradeoffs by Shoval et al. [14] that have not been previously tested on the scale of hundreds of millions of years of evolution. Ammonoid shell data on 990 genera is well-described by a square pyramid in morphospace. The five vertices of the pyramid may be interpreted as archetype morphologies optimal for a single task. Inferred tasks include shell economy, rapid growth, compactness and hydrodynamic efficiency. The vertices of the polygon do not change over the timescale of interest, as predicted in the case where the tasks stay more-or-less constant because they are dictated by biomechanical considerations. Moreover, the polygons and polyhedra in the theory are not necessarily due to phylogenetic history, but rather to convergent evolution to Pareto-optimal solutions. This agrees with the finding that after a mass extinction which removes almost all of the species, survivor species evolve to re-fill the same polygon as their ancestors. This approach may be used to infer biological tasks from data in other biological contexts.

## Methods

### Polygons, polyhedra and their statistical significance

We use the archetype analysis method SISAL [41] to compute the triangles in Figure 3. Since SISAL is only able to detect simplexes, we use PCHA [45] to find the three dimensional square pyramid, which is a polyhedron but not a simplex (more details in Additional file 1). To quantitate how well the triangle fits the data, we computed the t-value as in [14], the ratio between the area of the convex hull of the data and the and the area of the triangle found by SISAL. This t-ratio has a value t = 1 for a perfectly polygonal data. The t-ratio of the dataset is compared to a randomized dataset with the same number of genera, in which the parameters of each genera are randomly and independently chosen from their observed distributions. The fraction of times that 10,000 randomized dataset has a larger t-ratio than the real dataset is the p-value for the data polygonality [19].

### Statistical significance of triangle similarity

To compute similarity between two triangles, we computed the ratio between the area of intersection of the triangles and the area of their union. The larger this ratio, the more similar the triangles. To compute p-value the ratio was compared to that of 10,000 triangles whose vertices coordinate pairs were generated randomly from a uniform distribution on a rectangle. Note that the ratio is independent on the rectangle chosen. The p-value is the fraction of random triangle pairs with higher ratio than the measured one.

### Internal-volume to shell-volume performance function

We computed the internal to shell volume ratio numerically. Internal volume is computed by integrating along the spiral over the internal area added to the shell. Shell volume is computed by integrating the length of the curve added (part of a circumference of an ellipse), multiplied by the constant relative width of the shell from [47]. Note that, in order to obtain only the ratio of internal to shell volume, it is enough to compute the integrands themselves and it is not necessary to integrate over the spiral. More information can be found in Additional file 1.

### Growth performance function

The growth performance function is a simple model that penalizes small ammonoids according to the formula:

$$ P={\displaystyle \underset{0}{\overset{\infty }{\int }}\frac{1}{diam(t)}dt} $$

Where *diam(t)* is the minimal diameter of the ammonoid at a given time. Assuming that generation of shell material is proportional to body mass, this function is proportional to:

$$ P\sim \frac{W}{Ratio\left(D,W\right) \log (W)\left(1+\sqrt{W}\right)}. $$

Where Ratio(D,W) is the ratio of internal volume to shell volume as explained above. Details of this computation are in Additional file 1.

## Additional file

Additional file 1:
**Evolutionary tradeoffs, Pareto optimality and the morphology of ammonite shells – Supplementary Information.**

## Abbreviations

FF: Fransian/Femennian (mass extinction)
DM: Devonian/Missisipian (mass extinction)
PT: Permian/Triassic (mass extinction)
PCHA: Principal Convex Hull Analysis (algorithm)
SISAL: Simplex Identification via Split Augmented Lagrangian (algorithm)
MYA: Million years ago
