# Supplementary material for: Evolutionary tradeoffs, Pareto optimality and the morphology of ammonite shells
Source: BMC Syst Biol. 2015 Mar 7;9:12. doi: 10.1186/s12918-015-0149-z (PMC4404009; doi:10.1186/s12918-015-0149-z)
Supplement: Additional file 1: — Evolutionary tradeoffs, Pareto optimality and the morphology of ammonite shells – Supplementary Information. [file 12918_2015_149_MOESM1_ESM.docx]

**Evolutionary tradeoffs, Pareto optimality and the morphology of ammonite shells – Supplementary Information**

*Avichai Tendler, Avraham Mayo, Uri Alon*

*Dept. Molecular cell biology. Weizmann Institute of Science, Rehovot Israel 76100*

**Table of Contents**

1. **A triangle fits the data better than a W=1/D constraint**
2. **Archetype analysis algorithm**
3. **Computation of shell and internal volume**
4. **Construction of growth rate performance function**
5. **Square pyramid can be visualized via 2 dimensional slices**
6. **Large S ammonoids are approximately spherical**
7. **A triangle fits the data better than a W=1/D constraint**


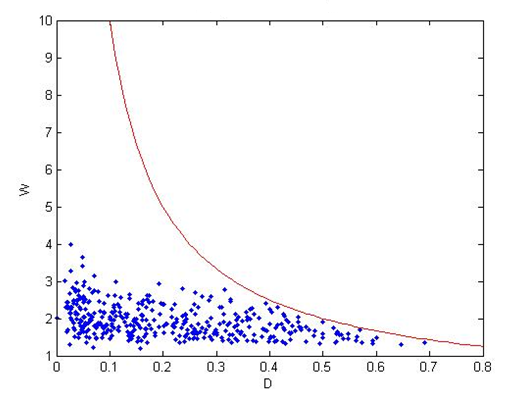


Fig S1. Ammonoid shell data (points) and the W=1/D hyperbola, to the right of which are shells with non-overlapping whorls.

The distribution of shell morphology is suggested in the main text to lie in a triangle, and in many previous studies to be instead constrained by the hyperbola W=1/D. We want to decide which hypothesis is in more agreement with the data. We use ammonoids between the DM and PT mass extinctions, for which we have the largest amount of data [1]. Intuitively, it is clear from Fig S1 that the line W=1/D does not describe the data very well, mainly due to the empty area in the W=3-4 region. Let us give a more quantitative measure for this intuition.

We want to understand the area capture by the data relative to the total volume under the curve. Of course, the area under the curve is infinite so we must take some restrictions. First, we take the geometric constraint and consider only the region W>1. The area is still infinite, and we restrict it further by bounding the region according to the existing D values. In order to avoid infinite area, we take the minimal nonzero D (0.002) as our lower limit, and the maximal D (0.69) as an upper limit. We compute the area of the bounded region:

$$V_{t}=\int_{D_{\min}}^{D_{\max}} \frac{dx}{x}-(D_{\max}-D_{\min})=5.15$$

Let us compare this to the area covered by the data points, which we approximate by the convex hull area which is 0.98. The volume ratio capture by the data is then the quotient 0.98/5.15= 0.19=19%.

In the triangle hypothesis, on the other hand, we obtain a triangle area of 0.89 and a ratio of 1.1, which is closer to 1. Note that the triangle does not necessarily contain all the data points, hence the ratio is larger than 1.

1. **Archetype analysis algorithm**

We use the principal convex hull (PCHA) archetype analysis algorithm of [2]. This algorithms provides an optimal polytope with a given number of vertices, in the sense that the sum of square error of data points outside the polytope is minimal. There is a restriction on the polytope, its vertices should be approximately convex combination of data points, where $\delta$ is the approximation parameter. To be more precise, each vertex should be a combination of data points with positive coefficients which sum to $1-\delta<s<1+\delta$, note that $s=1$ is the definition of being in the convex hull of the data points.

To choose $\delta$ we used a heuristic. The question is how well the data is sampled, for a perfectly sampled data one should take $\delta=0$, because the vertices are data points. To estimate how well the data is sampled we assume that data is uniformly sampled in the polytope. To demonstrate this, take for simplicity the d-dimensional cube $[0,1]^{d}$ sampled n times. The sampled point closest to (0,..,0) will have coordinates with values approximately $\frac{1}{n^{1/d}}$. Since we have about 300 data points in each period, we obtain the heuristics$\delta=0.06$ for 2-dimensional data and $\delta=0.16$for 3-dimensional data. These values are used throughout; changes of $\delta$ are insignificant for most of the results.

1. **Computation of shell and internal volume**

Fig S2: Schematic of shell shape for shells with overlapping whorls (left) and non-overlapping whorls (right).

Since our computation differs from that of [3], we describe the way we compute the internal volume to shell volume ratio.

Raup's approach is explained in [4], using an approximate volume equation of Moseley [5] to derive the ratio. We improve accuracy by numerically computing the internal volume to shell volume ratio.

Before we start with the computation, let us consider the simplest case W>1/D which can be solved analytically. In this case, cross-sections of the shell are simply disks (we take S=1 for now), see the Fig S2 on the right. To get the internal volume we integrate the area of over the entire spiral:

Where $V_{I}$ is the internal volume, $\theta$ is the expansion angle, $S$is the surface area in a given angle, $r$ is the distance from the center, is the internal length at a given $r$ and $\theta$ and $r_{average}$ is the average distance from the center.

The shell material volume is given by the length of the generating curve, also integrating over the spiral, using the same r averaging argument we get:

Here $V_{S}$ is shell volume, $C$is the ratio between shell width and radius calculated by [6] and is a length parameter of the generating curve.

In order to obtain the ratio we don't have to do the actual integration over $\theta$, which cancels, substituting the value of [6] for C , we obtain the ratio:

$$Ratio=\frac{1}{2C}=\frac{1}{2\cdot0.0772}=6.47$$

This also gives a feeling for on the error magnitude of Raup's calculations in [3], which gives 5.74 here.

The overlapping case also follows the above formulas, but we do not solve it analytically but rather numerically. The internal volume integral is now over a disk with a smaller disk cut removed, this is done because the removed circle was already counted in the previous round of the spiral. Similarly, the shell volume is computed using a circle with other circle cut removed, again, because the circle cuts were counted at the last round. In both cases, the $r_{average}$ trick will no longer work because the symmetry is now lost.

Clearly, in order to obtain the ratio it is enough to compute the cuts and we do not have to integrate over the spiral $\theta$ (this is still similar to the previous simpler case).

1. **Construction of growth rate performance function**

Fig S3: Definition of diameters of the ammonoid shell.

The function we consider in the main text was constructed to penalize the ammonoid for remaining small for too long. The penalty is given by:

$$P=\int_{0}^{\infty} \frac{1}{diam(t)}dt$$

Where $diam(t)$ is the minimal diameter of the ammonoid at a given time. In order to compute the integral we assume that the rate of generating shell material is proportional to the internal volume of the ammonoid. Under this assumption we can switch from integrating over time to integrating over angle. Up to a proportion constant related to the volume-specific shell production rate, we find:

$$P=\frac{1}{Ratio(D,W)}\int_{0}^{\infty} \frac{1}{diam(\theta)}d\theta$$

Here $Ratio$ denotes the volume to surface ratio computed above. To compute the integral, note that the radius of the ammonoid at a given time is $R(t)=R_{0}W^{\frac{\theta}{2\pi}}$, hence the minimal diameter is $diam(t)=R_{0}(\frac{1}{W}+\frac{1}{\sqrt{W}})W^{\frac{\theta}{2\pi}}$ (Fig S3). The square root results from the fact that the minimal diameter intersects the outer shell at two points whose angle is θ-2π and θ-π, resulting in radii that are 1/W and 1/sqrt(W) of the maximal shell radius. Computing the integral over $\theta$, we find (up to constants):

$P=\frac{W}{Ratio(D,W)\log(W)(1+\sqrt{W})}$ .

1. **Square pyramid can be visualized via 2 dimensional slices**


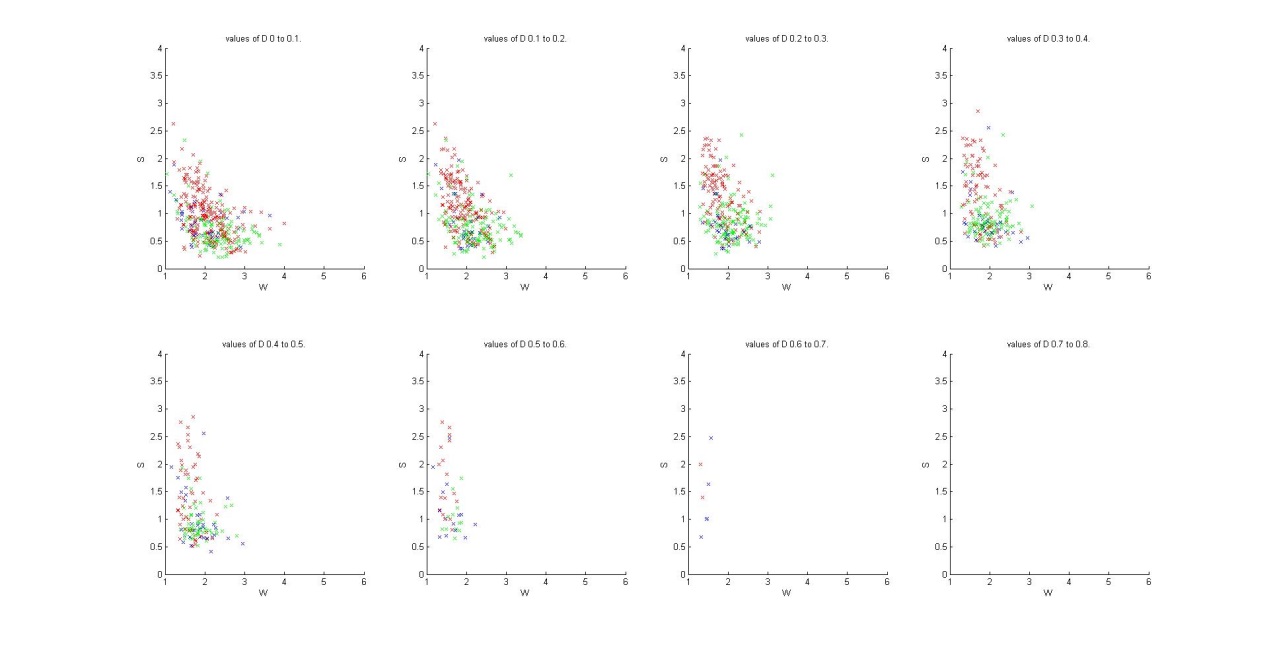


Fig S4: Slices of the 3D shell data at different values of D suggest a square pyramid-like arrangement of the data.

There are different approaches to understand higher dimensional geometries. A standard direction is dimensionality reduction techniques such as PCA, and another method which works in relatively lower dimensions is looking at projections on different axes.

One conceptual problem with dimensionality reductions and projections, is that these methods causes an inherent loss of information, because a 2D space is inspected instead of higher dimensional space.

Here we use a different method to understand geometries in dimensions slightly larger than 2. On the contrary to the methods discussed above, the method theoretically keeps all the information. The idea is that a shape in 3D (say), can be found by dividing to "slices" in one dimension and looking at the now 2D slices which can be analyzed. The final results are similar to that of a CT scan.

In the case of ammonoid data, we used slices over the D axis, with interval of width 0.1. The slices are shown in W-S plane in Fig S4. For small values of D, the slice obtained is approximately a right triangle. When increasing D, the W leg of the triangle increases, until only the S leg – a line –remains.

The information on the slices should be, in, principle, enough to determine the 3D geometry. In our case, the shape corresponding to the slices described above is a square pyramid. The apex of the pyramid has high W and low S and D values. The base of the pyramid has low W and is roughly parallel to the D-S plane. By this "CT scan" method, the square pyramid was discovered originally in this study.

In the main text we mention that when S becomes larger, the ammonoid shell tends to be more spherical. Here we demonstrate this statement.


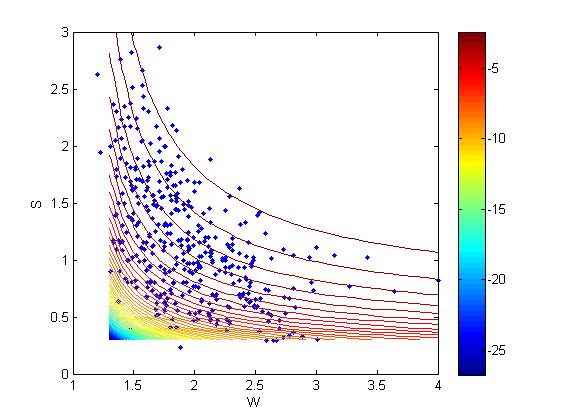

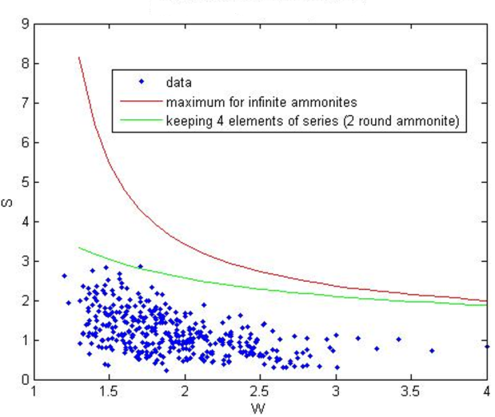


C

B

A

Figure S5. Measure of shell sphericity. (A) Schematic morphology of shell, with opening length normalized to one. (b) Contours of shell sphericity function $f\left( x \right)=-(\frac{L}{H}+\frac{H}{L})$, the length and height are computed in the main text. (c) Contours of perfect sphericity measure when shells have infinitely many whorls (red) or only four whorls (green). The latter suggests that high S ammonoids approach sphericity. [did I understand?]

To measure the sphericity of the shell, we compute whether the ratio length/height is close to 1 (see figure). To simplify the computation we assume that the shell lies on the curve W=1/D, but the ideas holds for general shell shapes. Let us scale everything as in the figure such that the length of the last spiral is 1. In this case the height is simply H=S, and the length is the geometric sum:

$$L=1+\frac{1}{\sqrt{W}}+\frac{1}{W}+\frac{1}{W^{3/2}}+...=\frac{\sqrt{W}}{\sqrt{W}-1}$$

Note that the figure in [7], as well as the data from [1] that we use, reveals that S=H<L, hence to obtain sphericity favors larger S for a given W. In other words, practically, H/L can approach 1 only from below.

We seek a measure for sphericity, such that the shell is more spherical the closer H/L is to one. Note that we want our measure for sphericity to preserve under inversion. In other words, a ratio of 1/2 is equivalent to a ratio of 2. Hence it is a function of the form f(x+1/x). We want the maximum to be f(1) and the function to be monotonic while going away from 1. The contours of our function look like the contours of –(x+1/x) where x=H/L is the ratio. This function and its maximum contour is plotted in Fig S5b Note that on the contrary to the FigS5c (red contour), ammonoids in nature can indeed be spherical and achieve the maximum of this function. The reason is that in nature the geometric sum in the definition of L is not infinite. Let us give the geometric series only a bounded number of elements. This cut of the sum will influence more in the region of small W. This is demonstrated in the green contour of Fig S5c: if we keep only 4 elements of the series, the data ammonoids with larger S will be spherical.

References

1. Saunders W. B, Work D. M: The evolutionary history of shell geometry in Paleozoic ammonoids. Paleobiology 2004, 30:19–43.
2. Mørup M, Hansen LK. Archetypal analysis for machine learning and data mining. Neurocomputing. 2012; 80:54–63.
3. Raup DM. Geometric analysis of shell coiling; coiling in ammonoids. J Paleontol. 1967;41:43–65.
4. Raup DM, Chamberlain JA. Equations for volume and center of gravity in ammonoid shells. J Paleontol. 1967;41:566–74.
5. Moseley H (1838) On the Geometrical Forms of Turbinated and Discoid Shells. *Philosophical Transactions of the Royal Society of London* 128:351–370.
6. Trueman AE. The ammonite body-chamber, with special reference to the buoyancy and mode of Life of the living ammonite. Q J Geol Soc. 1940;96:339–83.
7. Dommergues J-L, Montuire S, Neige P. Size patterns through time: the case of the Early Jurassic ammonite radiation. Paleobiology. 2002;28:423–34.
